# The core clock gene, Bmal1, and its downstream target, the SNARE regulatory protein secretagogin, are necessary for circadian secretion of glucagon-like peptide-1

**DOI:** 10.1016/j.molmet.2019.11.004

**Published:** 2019-11-21

**Authors:** Andrew D. Biancolin, Alexandre Martchenko, Emilia Mitova, Patrick Gurges, Everan Michalchyshyn, Jennifer A. Chalmers, Alessandro Doria, Josyf C. Mychaleckyj, Alice E. Adriaenssens, Frank Reimann, Fiona M. Gribble, Manuel Gil-Lozano, Brian J. Cox, Patricia L. Brubaker

**Affiliations:** 1 Department of Physiology, University of Toronto, Toronto, ON, Canada; 2 Department of Medicine, Harvard Medical School, Boston, MA, USA; 3 Research Division, Joslin Diabetes Center, Boston, MA, USA; 4 Center for Public Health Genomics, University of Virginia, Charlottesville, VA, USA; 5 Wellcome Trust-MRC Institute of Metabolic Science, University of Cambridge, Cambridge, UK; 6 Department of Obstetrics and Gynecology, University of Toronto, Toronto, ON, Canada; 7 Department of Medicine, University of Toronto, Toronto, ON, Canada

**Keywords:** Bmal1, Circadian, GLP-1, L-cell, Secretagogin, Secretion

## Abstract

**Objectives:**

The incretin hormone glucagon-like peptide-1 (GLP-1) is secreted from intestinal L-cells upon nutrient intake. While recent evidence has shown that GLP-1 is released in a circadian manner in rats, whether this occurs in mice and if this pattern is regulated by the circadian clock remain to be elucidated. Furthermore, although circadian GLP-1 secretion parallels expression of the core clock gene Bmal1, the link between the two remains largely unknown. Secretagogin (Scgn) is an exocytotic SNARE regulatory protein that demonstrates circadian expression and is essential for insulin secretion from β-cells. The objective of the current study was to establish the necessity of the core clock gene *Bmal1* and the SNARE protein SCGN as essential regulators of circadian GLP-1 secretion.

**Methods:**

Oral glucose tolerance tests were conducted at different times of the day on 4-hour fasted C57BL/6J, Bmal1 wild-type, and Bmal1 knockout mice. Mass spectrometry, RNA-seq, qRT-PCR and/or microarray analyses, and immunostaining were conducted on murine (m) and human (h) primary L-cells and mGLUTag and hNCI-H716 L-cell lines. At peak and trough GLP-1 secretory time points, the mGLUTag cells were co-stained for SCGN and a membrane-marker, ChIP was used to analyze BMAL1 binding sites in the *Scgn* promoter, protein interaction with SCGN was tested by co-immunoprecipitation, and siRNA was used to knockdown *Scgn* for GLP-1 secretion assay.

**Results:**

C57BL/6J mice displayed a circadian rhythm in GLP-1 secretion that peaked at the onset of their feeding period. Rhythmic GLP-1 release was impaired in Bmal1 knockout (KO) mice as compared to wild-type controls at the peak (p < 0.05) but not at the trough secretory time point. Microarray identified SNARE and transport vesicle pathways as highly upregulated in mGLUTag L-cells at the peak time point of GLP-1 secretion (p < 0.001). Mass spectrometry revealed that SCGN was also increased at this time (p < 0.001), while RNA-seq, qRT-PCR, and immunostaining demonstrated Scgn expression in all human and murine primary L-cells and cell lines. The mGLUTag and hNCI-H716 L-cells exhibited circadian rhythms in Scgn expression (p < 0.001). The ChIP analysis demonstrated increased binding of BMAL1 only at the peak of Scgn expression (p < 0.01). Immunocytochemistry showed the translocation of SCGN to the cell membrane after stimulation at the peak time point only (p < 0.05), while CoIP showed that SCGN was pulled down with SNAP25 and β-actin, but only the latter interaction was time-dependent (p < 0.05). Finally, *Scgn* siRNA-treated cells demonstrated significantly blunted GLP-1 secretion (p < 0.01) in response to stimulation at the peak time point only.

**Conclusions:**

These data demonstrate, for the first time, that mice display a circadian pattern in GLP-1 secretion, which is impaired in Bmal1 knockout mice, and that Bmal1 regulation of Scgn expression plays an essential role in the circadian release of the incretin hormone GLP-1.

## Highlights

• Murine GLP-1 secretion is circadian and is regulated by the clock gene Bmal1.
• The regulatory SNARE protein, secretagogin, is expressed in all intestinal L-cells.
• Secretagogin expression is circadian and regulated by BMAL1 in intestinal L-cells.
• Secretagogin translocates to the cell membrane upon L-cell stimulation.
• Secretagogin knockdown impairs rhythmic secretion of glucagon-like peptide-1.

## 1 Introduction

Circadian rhythms act as an anticipatory mechanism preparing organisms for the constant 24-hour light–dark cycle [1,2]. The main zeitgeber (ZT), light, entrains a network of clock genes in the suprachiasmatic nuclei of the hypothalamus, where these rhythms are orchestrated [1,3]. At the molecular level, these rhythms are generated by heterodimerization of the core clock protein BMAL1 with CLOCK and subsequent binding to E-box promoter elements to stimulate the transcription of *Period* (*Per*) 1–3 and *Cryptochrome* (*Cry*) 1–2. The expression of Per and Cry, in turn, represses *Bmal1* and *Clock*, completing the transcriptional feedback loop. The clock genes are known to be expressed in all nucleated mammalian cells, both centrally and in peripheral tissues, and are estimated to drive the rhythmic expression of approximately 43% of all protein-encoding genes [1,4].

Although light is the strongest ZT, nutrient intake can also synchronize peripheral metabolic tissues. The gastrointestinal tract, β-cell, liver, skeletal muscle, and adipose tissue [[5], [6], [7], [8], [9], [10], [11], [12], [13], [14]] have been shown to exhibit endogenous circadian rhythmicity, ultimately coordinating metabolic homeostasis with the 24-hour feeding–fasting cycle. In the well-characterized β-cell, insulin exhibits a diurnal rhythm in secretion and this pattern in insulin release is more pronounced when nutrients are delivered orally rather than intravenously [15]. This implicates temporal incretin secretion as an essential link between nutrient ingestion and deposition through the upregulation of glucose-stimulated insulin secretion.

It was previously reported that the enteroendocrine incretin hormone glucagon-like peptide-1 (GLP-1) is secreted in a circadian manner from enteroendocrine L-cells in rats and humans [[16], [17], [18], [19], [20]]. Although circadian GLP-1 secretion has been extensively tested in rat models using physiological disruptors such as constant light and obesogenic feeding [16,18], the lack of appropriate genetically modified animals has precluded determination of the role of the molecular clock in diurnal GLP-1 secretion. Circadian activity has also been shown in the murine (m) GLUTag and human (h) NCI-H716 L-cell lines, which exhibit cell-autonomous rhythmic patterns in *Bmal1*, with GLP-1 secretion paralleling *Bmal1* expression [16,17,21]. Furthermore, suppression of *Bmal1* with palmitate in mGLUTag L-cells is associated with dampened GLP-1 release, while primary intestinal cultures generated from *Bmal1* KO mice also demonstrate decreased GLP-1 secretion [18,21]. Nonetheless, the molecular mechanism linking Bmal1 expression to circadian GLP-1 secretion remains largely unknown.

Interestingly, impaired GLP-1 secretion has been observed in both cell and animal models of SNARE deficiency. The SNARE proteins mediate fusion of the secretory granule to the cell membrane, enabling exocytosis of the granule contents [22,23] and, indeed, the SNARE proteins, VAMP2, SYNTAXIN1A, and SYNAPTOTAGMIN-7, have been demonstrated to play essential roles in GLP-1 secretion [[24], [25], [26]]; however, it is uncertain if these proteins regulate secretion in a temporal manner. Evidence from α- and β-cells suggests that SNAREs and their accessory regulators exhibit rhythmic expression [27,28]. Secretagogin (SCGN), a SNARE-regulatory protein [[29], [30], [31]], has been identified as rhythmic in these cell types and has been shown to be essential for insulin secretion from β-cells [27,28,30,32,33]. SCGN is a calcium-binding protein that interacts with the core SNARE protein SNAP25 and β-actin in β-cells, both of which are also known to be involved in GLP-1 secretion by L-cells [24,30,32,34]. Given these similarities between β-cells and L-cells, SCGN was identified as a potential target linking circadian *Bmal1* expression to GLP-1 secretion.

Herein, for the first time, we define a circadian rhythm in GLP-1 secretion in mice, which is dependent on the core clock gene *Bmal1*. We also report that *Scgn* is expressed in intestinal L-cells, where it exhibits circadian expression under the transcriptional regulation of BMAL1. This drives a subsequent time-dependent recruitment of SCGN toward the L-cell membrane that in turn facilitates circadian secretion of GLP-1. When taken together, we identified a novel regulator of circadian GLP-1 secretion, which could have implications for time-sensitive treatments as well as the potential for SNAREs as targets for type 2 diabetes therapies.

## 2 Methods

### 2.1 Animals

Male and female C57Bl/6J mice and Bmal1^+/−^ mice were purchased from Jackson Laboratories (Bar Harbor, ME, USA). The Bmal1^+/−^ mice were bred and genotyped according to the recommended protocol to generate sex-, age-, and littermate-matched wild-type (WT) and KO animals. The mice had free access to water and a regular chow diet (Teklad) for the duration of the study and were allowed to acclimate for one week to the 12-hour light, 12-hour dark (lights on at 06:00 or Zeitgeber Time (ZT) 0) and constant room temperature conditions at the animal facility before use. All experimental procedures were approved by the Animal Care Committee of the University of Toronto.

### 2.2 Oral glucose tolerance tests

Oral glucose tolerance tests (OGTTs) were conducted on 4-hour fasted mice with their basal blood glucose obtained prior to the administration of an oral gavage of glucose at a concentration of 5 g/kg of body weight [25]. OGTTs were conducted on the C57Bl/6J mice at ZT2, 6, 10, 14, 18, and 22. The nighttime studies were carried out under a red light; two tests were conducted on most animals with a one-week recovery interval. Bmal1 WT and KO mice were tested at the trough (ZT2) and peak (ZT14) time points of GLP-1 secretion established in the C57Bl/6J mice. Blood was collected from the tail vein at 0 min and then 10 and 60 min after the oral gavage to measure the glucose using a OneTouch meter (LifeScan, Burnaby, BC, Canada) and the plasma GLP-1 and insulin levels using a MesoScale Discovery (MSD) assay for the total GLP-1.

### 2.3 Cell culture

Male mGLUTag cells were used as a model of intestinal L-cells due to their close representation of in vivo GLP-1 secretion [16,21]. The cells were grown in DMEM with 25 mmol/L glucose and 10% FBS [16,21]. Male hNCI-H716 cells were used as a human L-cell model because they respond to known GLP-1 secretagogues [17,35]. They were grown in suspension in cell culture flasks with RPMI 1640 medium containing 10% FBS and 100 U/ml penicillin/streptomycin. For the experiments, hNCI-H716 cells were plated onto cell culture plates coated with 0.5 g/ml Corning Matrigel (Thermo Fisher Scientific, Waltham, MA, USA).

### 2.4 In vitro synchronization

For all of the circadian experiments, the cells were synchronized using a previously established protocol [[16], [17], [18],^21^]. In brief, the cells were grown for two days from the last split and starved in an appropriate media containing 0.5% FBS for 12 h to induce quiescence. The cells were then synchronized with 20 μM forskolin (Sigma–Aldrich, Oakville, ON, Canada) for 1 h, after which the media was changed to growing media for up to 48 h. While previous reports suggest that certain synchronizing agents generate more robust rhythms than others [36], forskolin was used as a synchronizer because it has been previously shown to elicit a strong circadian response in immortalized L-cells [[16], [17], [18],^21^]. Another established synchronizer, 30% FBS, was also tested with the mGLUTag L-cells, but resulted in significant cell death after 24 h (unpublished data).

### 2.5 Human and murine primary cell RNA sequencing

Fluorescent-assisted cell sorting (FACS) of L-cells, either via the expression of the fluorescent reporter Venus in transgenic mice or staining with fluorescent antibodies in human tissue isolates, was described previously [37]. RNA isolation and sequencing is described in [37,38] and the data were deposited in the NCBI GEO repository (human, GSE114853; mouse, GSE114913). The studies were conducted in accordance with the principles of the Declaration of Helsinki and good clinical practice. Human ethical approval was provided by Cambridge Central and South Research ethics committees (Ref: 09/H0308/24, 16/EE/0338, and 15/EE/0152), the Inserm ethics committee, and Agence de la Biomédecine (Ref: PFS16-004). The animal research was regulated under the Animals (Scientific Procedures) Act 1986 Amendment Regulations 2012 and conducted following an ethical review by the University of Cambridge Animal Welfare and Ethical Review Body.

### 2.6 hNCI-H716 cell RNA sequencing

Total RNA was extracted using an RNeasy Plus Mini Kit (Qiagen) according to the manufacturer's instructions and the samples were tested using an Agilent BioAnalyzer to assess their integrity (visual electropherogram inspection and RIN score). Sequencing libraries were prepared using an Illumina TruSeq RNA Library Prep Kit v2 with polyA selection and run on an Illumina HiSeq 2500 using Rapid Flow Cell v2 at the Harvard University's Bauer Sequencing Core, with 76 cycle paired end reads as part of a larger multiplex group of barcoded RNA sample libraries. FASTQC v0.11.8 detected no unexpected conditions in the resulting reads. The reads (minus the last base) were aligned to human genome assembly 38 and gene models from GENCODE v28 primary assembly using STAR 2.6.0 [39]. Gene read quantification was performed using RSEM v1.3.1 [40]. Genes with very low total expression (total expected counts < 1, low + high conditions) were removed and the remainder normalized using the trimmed mean of M-values method [41] as implemented in the edgeR R package. The log_2_ counts per million (CPM) were estimated with a prior default of 0.25 CPM per gene. The data were deposited in the NCBI GEO repository (human, GSE136369).

### 2.7 Microarray analysis

The RNA was extracted, reverse-transcribed, and subjected to microarray analysis at the Ontario Cancer Institute Genomics Centre (Toronto, ON, Canada) using a mouse WG-6 V2 Illumina BeadChip as previously reported [16]. Gene ontology enrichment data were obtained from the Walter and Eliza Hall Institute of Medical Research bioinformatics resource on August 20, 2019 (http://bioinf.wehi.edu.au/software/MSigDB/mouse_c5_v5p2.rdata). The data were deposited in the NCBI GEO repository (human, GSE136573).

### 2.8 Gene expression analyses

Total RNA from FACS-sorted cells [37] was isolated using a Microscale RNA Isolation Kit (Ambion) and reverse transcribed according to standard protocols. Quantitative RT-PCR was performed with a 7900 HT Fast Real-Time PCR system (Applied Biosystems). The PCR reaction mix consisted of first-strand cDNA template, appropriate TaqMan probe/primer mix, and PCR Master Mix (Thermo Fisher Scientific). The expression of *Scgn* was compared with that of *Actb* measured on the same sample in parallel on the same plate, demonstrating a CT difference (ΔCT). The mean, standard error, and statistical analyses were performed on the ΔCT data and only converted to relative expression levels (2 ˆ ΔCT) for presentation in the figures.

The total RNA collected from cell lines using a Paris Kit (Thermo Fisher) was reverse-transcribed using 5X All-In-One Reverse Transcriptase MasterMix (Applied Biological Materials, Richmond, BC, Canada), and quantitative RT-PCR was conducted using a TaqMan Fast Mix Gene Expression Assay with primers (Thermo Fisher Scientific) as listed in Table S1. Gene expression was calculated using the ΔΔCt method [42]. *H3f3a* (mGLUTag) and *Hist1h3a* (hNCI-H716) were used as control genes as they have been previously established to lack circadian rhythms [16,17,21].

### 2.9 Mass spectrometry

The mGLUTag cells were grown and synchronized as previously described, and the total protein was extracted at 8 and 20 h after cell synchronization using TRIzol reagent. The protein extract was analyzed via mass spectrometry at the SPARC BioCentre, Hospital for Sick Children (Toronto, ON, Canada). The data were analyzed using Scaffold proteome software and the DAVID functional annotation bioinformatics tool.

### 2.10 Immunoblotting analyses

Protein was collected using a Paris Kit, quantified by a Bradford assay, run on 10% polyacrylamide gel, and transferred onto polyvinylidene difluoride membranes. The membranes were blocked for 1 h in 5% skim milk in Tris-buffered saline with 0.1% Tween (TBS-T, Sigma–Aldrich). The blots were incubated overnight in skim milk TBS-T with antibodies as listed in Table S2 and washed in TBS-T. Following incubation with anti-rabbit IgG secondary antibodies (Cell Signaling, Danvers, MA, USA; Table S2) in skim milk TBS-T, the membranes were imaged with SignalFire Elite Enhanced chemiluminescent reagent (Cell Signaling) and visualized on a Kodak imaging system (Eastman Kodak Company, Rochester, NY, USA). When the membranes were re-probed, the blots were first stripped using Restore PLUS Western blotting stripping buffer (Thermo Fisher Scientific) and washed in TBS-T.

### 2.11 Immunofluorescence

Formalin-fixed, paraffin-embedded murine (UHN Pathology Services, Toronto, ON, Canada) and human (OriGene) ileal sections were dewaxed, rehydrated, and blocked in 10% normal goat serum (NGS)/PBS for 1 h and then incubated in 10% NGS/PBS with rabbit anti-Scgn (Cell Signaling) and mouse anti-GLP-1 (Abcam, Inc., Toronto, ON, Canada) antibodies for 1 h (Table S2), followed by incubation with Alexa Fluor 488- and 555-labeled secondary antibodies (Table S2) for 1 h. The immunostained cells were counted and compared based on the presence and/or absence of GLP-1 and SCGN co-staining, with the average percent distribution determined as the number of cells in each category divided by the total number of cells observed.

Cells for immunocytochemistry were grown on Falcon multi-chamber microscope slides. For translocation experiments, the cells were synchronized as previously described and then treated with 10^−7^ M glucose-dependent insulinotropic polypeptide (GIP, an established rodent L-cell secretagogue [[16], [17], [18],^21^]). Live cells were then incubated in 2.5 μg/μl of wheat germ agglutinin-Alexa Fluor 488 Conjugate (Thermo Fisher) in HBSS at 37 °C for 10 min. All of the cells were then fixed in 4% paraformaldehyde for 30 min at 37 °C, permeabilized with 0.1% Triton X-100 (Sigma–Aldrich) in PBS for 20 min, and incubated in 1% BSA for 30 min at 37 °C, followed by incubation with rabbit anti-Scgn antibody (Table S2). All of the cells were then incubated with Alexa Fluor 488-labeled secondary antibody for 1 h (Table S2). Pearson's correlation coefficient (PCC) was used to measure the colocalization of wheat germ agglutinin membrane staining and SCGN using the mean intensities of the green and red channels, respectively [43]; a value of zero represents probes that were not correlated with one another. Regions of interest were generated in an unbiased manner by outlining the cell membrane. PCC was calculated using NIS-Elements imaging software (Nikon Corporation).

The stained sections and cells were mounted in Vectashield mounting medium containing DAPI (Vector Laboratories, Burlington, ON, Canada). Imaging was conducted using a Nikon Swept Field confocal microscope and immunofluorescence analysis was performed with NIS-Elements Imaging.

### 2.12 Chromatin immunoprecipitation assay

Non-canonical E-boxes for BMAL1 [44] (CATG(T/C)G) were identified in the 5′ *Scgn* promoter at 672 bp (CATGCG), 1176 bp (CACGCG), and 1252 bp (CATGTG) upstream of the transcription start site. ChIP was conducted using a SimpleChIP Enzymatic Chromatin IP Kit with Magnetic Beads #9003 (Cell Signaling) per the manufacturer's instructions. In brief, cell protein was cross-linked to DNA with 37% formaldehyde for 10 min at room temperature, digested with micrococcal nuclease for 20 min at 37 °C, and incubated with BMAL1 antibody at 4 °C overnight (Table S2). The protein was precipitated using Protein G magnetic beads and the DNA was eluted from the beads using a Magnetic Separation Rack (Cell Signaling). The DNA was amplified by PCR using SYBR Green [45] with the listed primers (Table S3).

### 2.13 Co-immunoprecipitation

Cells were washed with HBSS and lysed with Cell Lysis Buffer (Cell Signaling). The protein (200 μg in 200 μl of lysis buffer) was incubated with 4 μl of anti-SNAP25 or anti-Scgn antibody (Table S2) overnight with rotation, incubated with Protein A Magnetic Beads (New England Biosystems) for 2 h with rotation, and washed with lysis buffer. The beads were then placed in 20 μl of 3X Reducing SDS Loading Buffer (Cell Signaling) and heated at 95 °C for 5 min. The protein effluent was collected using a Magnetic Separation Rack (Cell Signaling) and loaded directly onto a 10% polyacrylamide gel as previously described for immunoblotting of SCGN or β-actin, respectively (Table S3).

### 2.14 siRNA-mediated knockdown

In vitro knockdown studies utilized ON-TARGETplus siRNA for *Scgn* and ON-TARGETplus Non-targeting Pool scRNA as a control (Dharmacon Inc., Lafayette, CO, USA). The RNA constructs were used in combination with Dharmafect3 using a reverse-transfection protocol as recommended by the manufacturer (Dharmacon Inc.). To maintain knockdown in the synchronization experiments, the media for both starvation (0.5% FBS) and serum shock (10% FBS with forskolin) steps and all subsequent incubations included siRNA or scRNA in combination with Dharmafect3 as appropriate. Cells were then extracted for qRT-PCR or analyzed for GLP-1 secretion.

### 2.15 GLP-1 secretion assay

Cells were incubated for 2 h in DMEM with 0.5% FBS containing 10^−7^ M GIP or vehicle alone (control). As previously noted, GIP was used as a secretagogue due to its ability to reliably stimulate temporal GLP-1 release by rodent L-cells [[16], [17], [18],^21^]. Peptides in the media and cells were collected by reversed-phase adsorption using Sep-Pak cartridges (Waters Associates, Milford, MA, USA), and the GLP-1 levels were measured using a Total GLP-1 Radioimmunoassay Kit (Millipore, Etobicoke, ON, Canada). All of the data are expressed as percent secretion determined as the 100x the media content of the GLP-1 divided by the total (media + cells) GLP-1 content.

### 2.16 Statistical analyses

All data are expressed as mean ± SEM. The delta area under the curve (ΔAUC) was calculated using the trapezoidal rule for the changes in hormonal and glycemic responses from fasting over the entire 60-min study. In vivo circadian data were tested for significant 24-hour rhythms using CircWave software (www.hutlab.nl). In vitro rhythmic analyses enabling the identification of the significant period was calculated using MetaCycle [46,47] and curves were plotted using a damped sine feature with GraphPad Prism. All other figures were analyzed for significance via ANOVA (1-, 2-, or 3-way) followed by Tukey's test as appropriate. Differential expression analysis of the microarray and protein expression was performed using linear models through the R package limma.

## 3 Results

### 3.1 Circadian GLP-1 secretion is dependent on the core clock gene Bmal1

**Figure 1:**
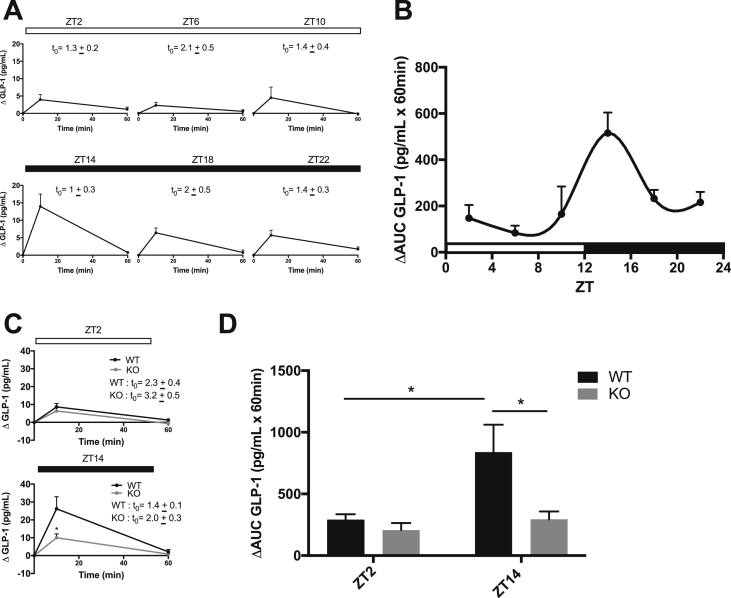
**Circadian GLP-1 secretion is dependent on the core clock gene Bmal1.** (**A-B**) OGTTs were conducted on 4-hour fasted C57Bl/6J mice at six time points throughout a 24-hour light (open bars)-dark (closed bars) cycle (ZT0 = 06:00), with individual plots for each time point represented in (**A**) and the 24-hour ΔAUC profile represented in (**B**). (**C-D**) OGTTs were performed on 4-hour fasted Bmal1 WT and KO mice at ZT2 and ZT14 with individual plots for each time point shown in (**C**) and the ΔAUC of each timepoint represented in (**D**). (**A** and **C**) t_0_ indicates the absolute fasting values for GLP-1 in pg/ml. n = 4–8 mice for all time points in all of the experiments. *p < 0.05.

To establish whether GLP-1 secretion follows a circadian rhythm in mice, 4-hour fasted C57Bl6/J mice were administered an identical oral glucose load at six time points throughout a 24-hour day. Fasting levels of GLP-1, insulin, and blood glucose varied slightly by time of day (Figures 1A and S1A). Therefore, to directly compare the L-cell secretory response to the same OGTT over the course of the day, the data were examined as the change from the basal levels. As expected, plasma GLP-1 increased at 10 min post oral gavage at all times of the day; however, the peak response was observed at ZT14, which aligns with the onset of the dark, the feeding period in mice (Figure 1A). Representation of the data as the ΔAUC generated a curve that significantly (p < 0.05) fit to a 24-hour rhythm, with a peak at ZT14 and a corresponding trough at ZT2 (Figure 1B). The plasma insulin and blood glucose concentrations also increased at all time points after the oral glucose load (Figure S1A).

Although we previously established that ex vivo adult mouse ileal crypt cultures generated from Bmal1 KO mice have impaired GLP-1 secretory capacity [21], time-dependent GLP-1 secretion had not yet been determined in vivo in these animals. We therefore conducted OGTTs at the established trough (ZT2) and peak (ZT14) of the GLP-1 secretion in 4 h-fasted Bmal1 KO compared to WT (control) mice. Consistent with their known impairment in pancreatic β-cell function [9], the KO mice demonstrated impaired insulin release at both time points (Figure S1B), with corresponding hyperglycemia (Figure S1C). The WT mice displayed greater GLP-1 secretion at ZT14 than ZT2, confirming the pattern observed in the C57Bl6/J mice. In contrast, the KO mice demonstrated a loss of the normal peak and trough rhythm, with markedly impaired GLP-1 release at the peak (ZT14) time point of secretion only (p < 0.05; Figure 1C–D). Taken together, these data establish a circadian pattern in GLP-1 secretion in the mouse that depends on the expression of the core clock gene *Bmal1*.

### 3.2 Identification of secretagogin as a potential regulator of circadian GLP-1 secretion

**Figure 2:**
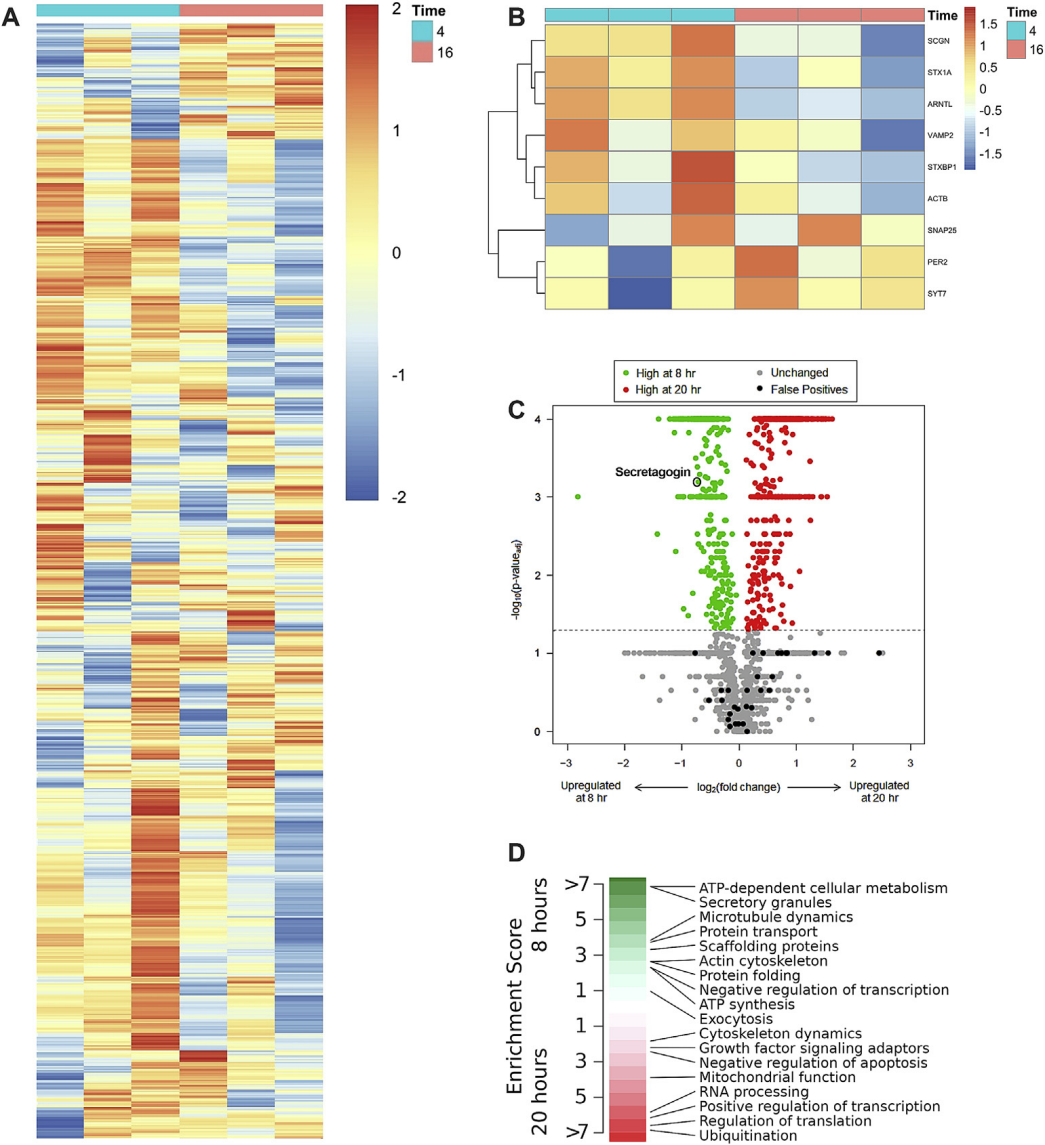
**Identification of potential targets regulating circadian GLP-1 secretion.** (**A**) Heat maps of GO transport vesicle and (**B**) selected L-cell secretory genes identified by microarray in synchronized the mGLUTag L-cells showing log_2_ fold-change between the two time points, 4 and 16 h (n = 3 for each time point). (**C**) Volcano plot of the mass spectrometry results of the synchronized mGLUTag L-cells at peak (8 h) and trough (20 h) of GLP-1 secretion (n = 3 for each time point). SCGN is indicated by the open circle. (**D**) Pathway enrichment analysis of protein clusters that were up regulated at each time point, with SCGN identified under the secretory granules cluster.

To identify targets linking the rhythmic expression of Bmal1 to the circadian secretion of GLP-1, a microarray analysis was conducted on the synchronized mGLUTag cells at the previously reported peak (4 h) and trough (16 h) of *Arntl* (Bmal1) mRNA expression [16,17] (see also Figure 4A,F). Although no genes were significantly downregulated, 13 genes were identified that were significantly upregulated (p < 0.05; Table S4), including the positive control *Arntl* (log_2_ fold-change = 1.103288, p < 0.05). Furthermore, 34 pathways relating to vesicle transport were significantly different between the two time points (p < 0.05; Table S5), including the GO transport vesicle (Figure 2A) and GO SNARE complex (Figure S2), which were both significantly upregulated at the 4-hour time point. The GO transport vesicle includes proteins that move cargo between the ER and Golgi or to the membranes for secretion, including the SNARE proteins identified in the GO SNARE complex. This group also includes transcripts for accessory proteins that facilitate vesicle/granule transport, such as *Scgn* and *Stxbp1*. A number of genes in this pathway that are essential for secretion from the L-cells were increased with *Bmal1* at the 4-hour time point (Figure 2B) as well as the transcript for *Scgn*. In addition, given the importance of the actin cytoskeleton in GLP-1 secretion [34] and a recently reported interaction between SCGN and β-actin [32], GO-actin cytoskeleton (Figure S3) was identified as a significantly upregulated pathway at the 4-hour time point (p < 0.05), which includes *Actb*, *Stx1b*, *Vamp2*, and *Snap25*. Collectively, these findings suggest possible roles for vesicle transport and/or SNARE proteins in the regulation of circadian GLP-1 secretion.

**Figure 3:**
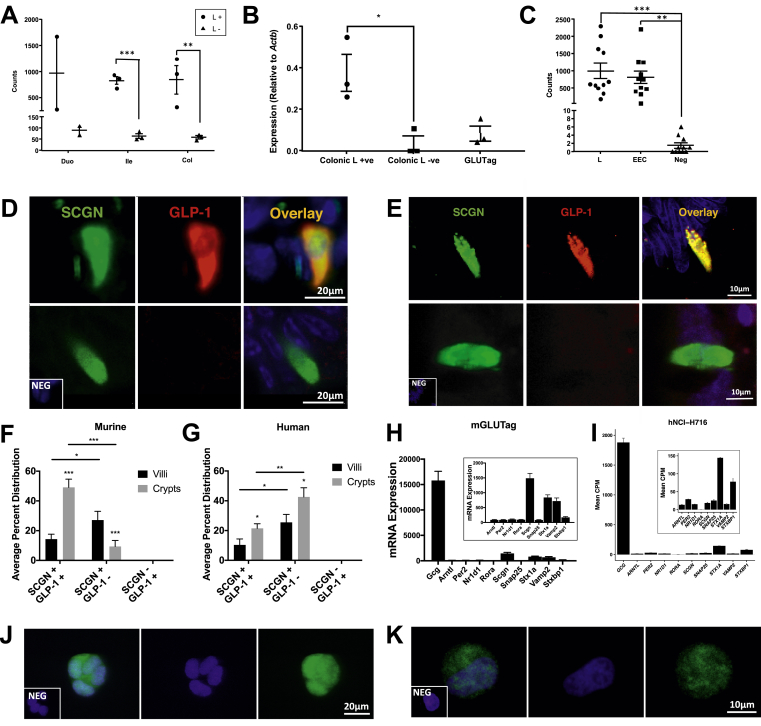
**Scgn is expressed in primary and immortalized murine and human L-cells.** (**A**) RNA-seq analysis of *Scgn* in murine *Gcg*-Venus L-cells and Venus-negative cells from duodenal, ileal, and colonic sections (n = 2–3 for each cell population). (**B**) RT-qPCR for *Scgn* in murine colonic Gcg-Venus L-cells, Venus-negative cells, and mGLUTag L-cells (n = 3 for each cell population). (**C**) RNA-seq analysis for *SCGN* in human jejunal L-cells, enteroendocrine cells, and non-endocrine intestinal epithelial cells (n = 11 for each cell population). (**D-G**) Immunostaining of murine (**D**) and human (**E**) ileal sections for SCGN and GLP-1 (representative images of SCGN^+^:GLP-1^+^ (top) and SCGN^+^:GLP-1^-^ (bottom) cells are shown). Cell count histogram for murine (**F**) and human (**G**) ileal SCGN and/or GLP-1 stained cells (n = 5 sections for each species; a total of ∼100 cells were counted per section). (**H-I**) Microarray analysis of mGLUTag cells (n = 6) (**H**) and RNA-seq analysis of hNCI-H716 cells (n = 2) (**I**) for proglucagon, clock, and SNARE protein transcripts. (**J-K**) Immunostaining of mGLUTag **(J**) and hNCI-H716 (**K**) L-cells for SCGN; DAPI shows the nuclear stain (representative images of n = 4 are shown). *p < 0.05, **p < 0.01, ***p < 0.001.

**Figure 4:**
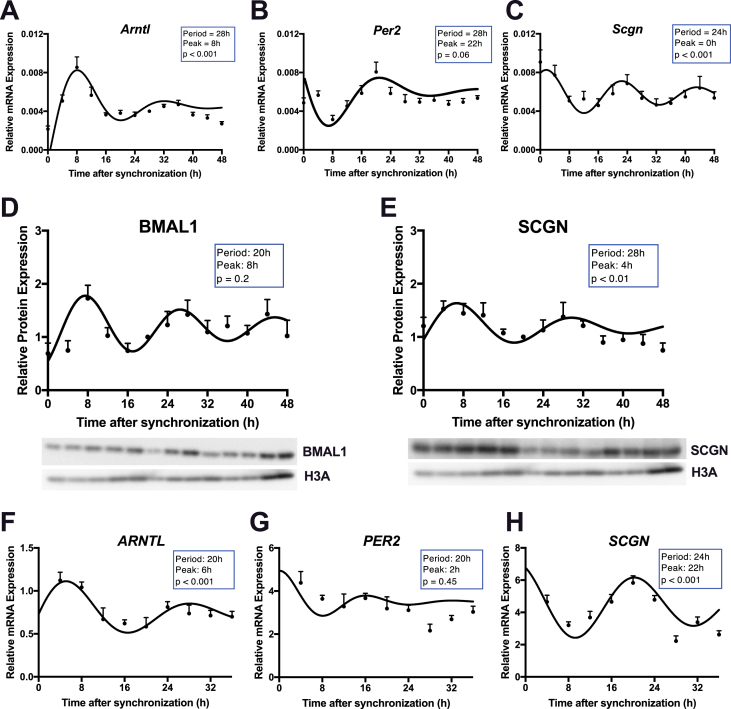
**Scgn expression is circadian in mGLUTag and hNCI-H716 L-cells.** (**A-C**) mRNA and (**D-E**) protein expression over 48 h in synchronized mGLUTag L-cells for *Bmal1* (**A**), *Per2* (**B**), *Scgn* (**C**), BMAL1 (**D**), and SCGN (**E**; n = 8, conducted as 4 replicates from each of 2 independent splits; representative blots are shown in (**D-E**)). **(F-G**) mRNA expression over 36 h in synchronized hNCI-H716 cells for *BMAL1* (**F**), *PER2* (**G**), and *SCGN* (**H**; n = 8, conducted as 4 replicates from each of 2 independent splits).

To further screen for targets mediating the temporal secretion of GLP-1, the proteome of the synchronized mGLUTag cells was studied at the peak (8 h) and trough (20 h) time points of GLP-1 secretion [16] (see also Figure 8E). Mass spectrometry analysis identified 2,050 proteins with a <5% false-positive detection rate, including 1,749 known proteins, with 333 proteins upregulated at the 8-hour time point and 414 proteins upregulated at the 20-hour time point. SCGN was identified as significantly upregulated (p < 0.001, Figure 2C) at the peak of GLP-1 secretion (8 h). Gene set enrichment analysis (Figure 2D) of the mass spectrometry data identified several pathways matching the microarray findings, including the upregulation of proteins related to secretory granules (including SGCN), protein transport, and the actin cytoskeleton (both including β-actin) at the 8-hour time point.

**Figure 5:**
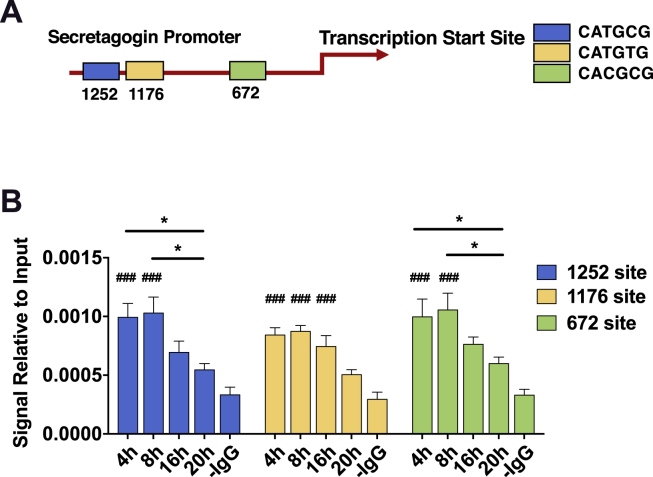
**Time-dependent binding of BMAL1 to the *Scgn* promoter in mGLUTag L-cells.** (**A**) Three noncanonical BMAL1 E-boxes were identified at 1252, 1176, and 672 base pairs upstream of the transcription start site. (**B**) ChIP analysis for the BMAL1 binding sites in the *Scgn* promoter at 4, 8, 16, and 20 h in synchronized mGLUTag L-cells (n = 6, conducted as 3 replicates from each of 2 independent splits). *p < 0.05; ###p < 0.001 vs negative control (-IgG).

**Figure 6:**
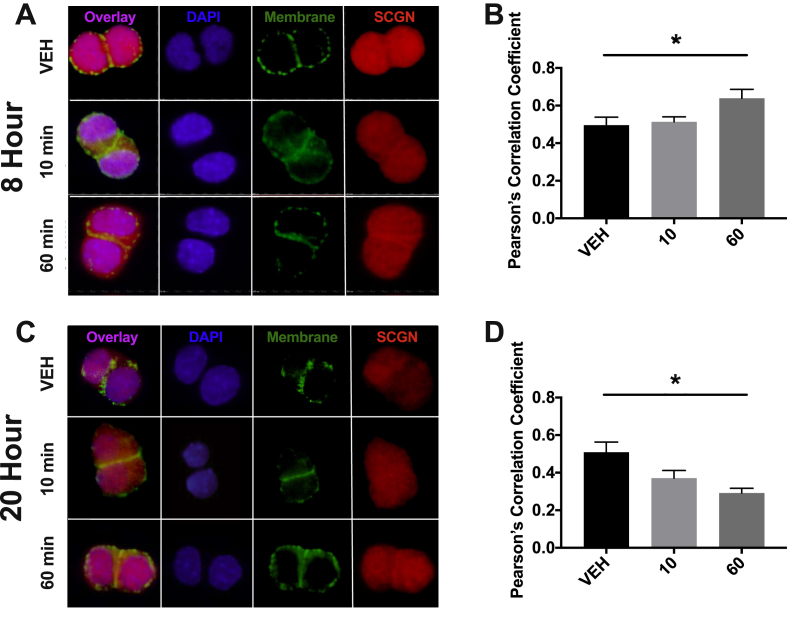
**Time-dependent recruitment of SCGN to the cell membrane in mGLUTag L-cells.** (**A-B**) 8 h and (**C-D**) 20 h after synchronization, mGLUTag L-cells were treated with 10^−7^ M GIP or vehicle for 0, 10, and 60 min, followed by staining for SCGN and a membrane marker (with wheat germ agglutinin). Co-localization of SCGN with the cell membrane was determined by Pearson's correlation coefficient. (n = 4, conducted as 2 replicates from each of 2 independent splits; representative images are shown). *p < 0.05.

**Figure 7:**
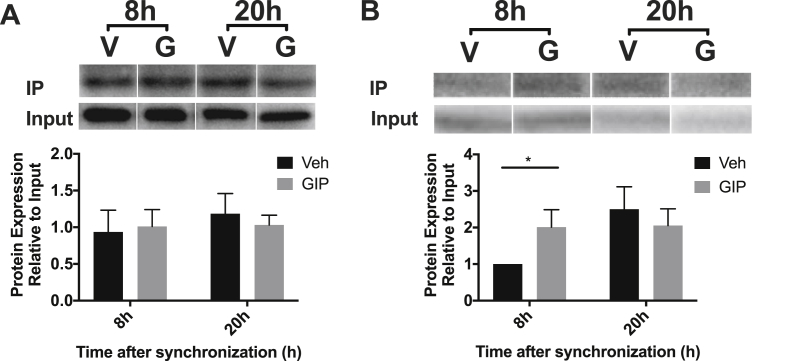
**Time-dependent interactions of SCGN with β-actin but not SNAP25 in mGLUTag L-cells.** mGLUTag L-cells were synchronized and then 8 or 20 h later treated with 10^−7^ M GIP or vehicle for 2 h. (**A**) Immunoprecipitation of SNAP25 and immunoblotting for SCGN. (**B**) Immunoprecipitation of SCGN and immunoblotting for β-actin. (n = 4, conducted as 2 replicates from each of 2 independent splits; representative blots are shown). *p < 0.05.

**Figure 8:**
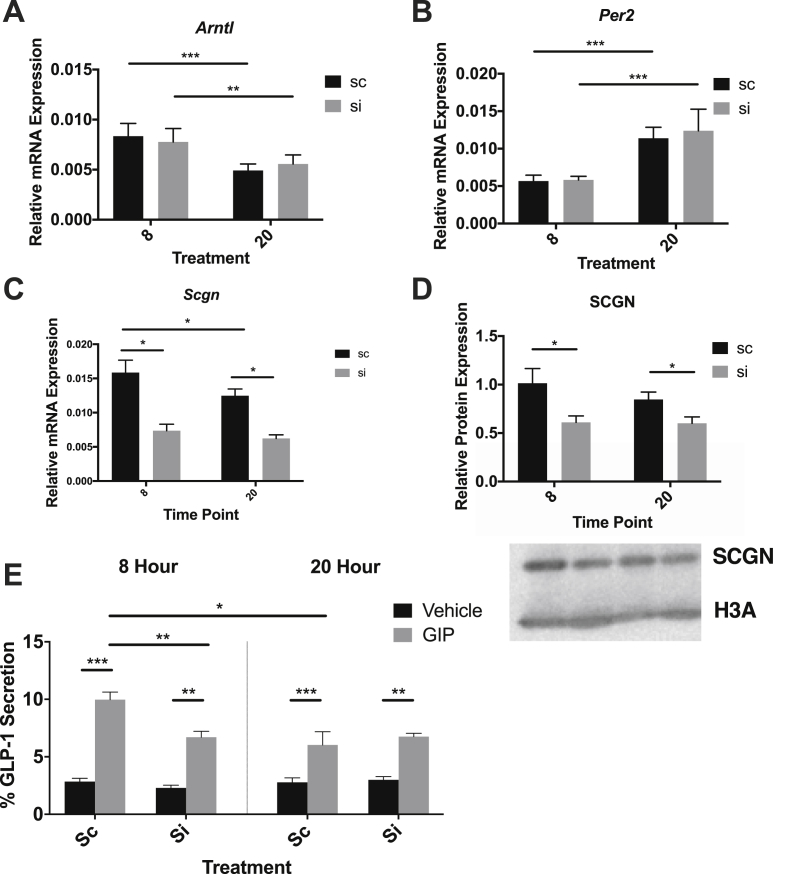
**Scgn is essential for peak GLP-1 secretion in mGLUTag L -cells.** mGLUTag L-cells were treated with scRNA or *Scgn* siRNA, synchronized and then 8 or 20 h later treated with 10^−7^ M GIP or vehicle for 2 h. The cells were then analyzed for *Bmal1* (**A**), *Per2* (**B**), *Scgn* (**C**), SCGN (a representative blot is shown) (**D**), and GLP-1 secretion (**E**) (n = 8, conducted as 4 replicates from each of 2 independent splits). *p < 0.05, **p < 0.01, ***p < 0.001.

RNA-seq analysis conducted on intestinal cells collected from murine *Gcg*-Venus mice revealed that *Scgn* was expressed at higher levels in L-cells in the ileum and colon than other intestinal epithelial cells (p < 0.001 and p < 0.01, respectively; Figure 3A). *Scgn* expression was also analyzed by RT-qPCR and compared between colonic L+/L-cells and mGLUTag cells, confirming that colonic L-cells express more secretagogin than non-L-cells (p < 0.05, Figure 3B). In addition, human jejunal L-cells were compared with enteroendocrine cells (EECs) and other intestinal epithelial cells for *SCGN* expression, with both L-cells and EECs expressing more secretagogin than the negative controls (p < 0.01–0.001, Figure 3C).

Murine (Figure 3D) and human (Figure 3E) ileal sections were also immunostained to assess the co-expression of secretagogin with GLP-1. Analysis of the percent distribution of SCGN^+^ and GLP-1^+^ cells (Figure 3F–G) revealed that SCGN was expressed in all of the GLP-1^+^ cells. SCGN was also identified in a significant number of GLP-1^-^ cells. However, the murine ileal samples had a higher percentage of SCGN^+^:GLP-1^+^ cells than SCGN^+^ GLP-1^-^ cells in the crypts (p < 0.001), whereas this distribution was reversed in human ileal tissue (p < 0.001).

To determine the expression of several genes of interest in our cell models, microarray and RNA-seq analyses were conducted on the mGLUTag (Figure 3H) and hNCI-H716 (Figure 3I) cells, respectively. Transcripts for *Scgn/SCGN* were expressed in both cell lines and in those for proglucagon (*Gcg*; the prohormone for GLP-1) and a variety of different known clock (*Arntl*, *Per2*, *Nr1d1*, and *Rora*) and SNARE (*Stx1a*, *Vamp2*, *Snap25*, *Scgn*, and *Stxbp1*) proteins. Immunostaining further demonstrated that SCGN was localized to both the nucleus and cytoplasm of the mGLUTag (Figure 3J) and hNCI-H716 (Figure 3K) cells. Collectively, these findings demonstrate the expression of secretagogin in the murine and human L-cell both in vivo and in vitro.

### 3.3 Scgn is expressed in a circadian manner in murine and human L-cells

To further investigate the rhythmic expression of secretagogin, mRNA was extracted from synchronized mGLUTag cells every 4 h for 48 h. The circadian expression of *Arntl* (p < 0.001; Figure 4A) and anti-phasic expression of *Per2* (p = 0.06; Figure 4B) was used as a positive control for the synchronization as previously reported [16,21]. *Scgn* mRNA expression peaked at 0 h, with a period of 24 h (p < 0.001; Figure 4C), consistent with a circadian rhythm. To further analyze the rhythmic properties of secretagogin, protein was also collected and analyzed for the circadian expression of BMAL1 (Figure 4D) and SCGN (Figure 4E). Although the pattern in BMAL1 did not reach significance, SCGN demonstrated a significant circadian rhythm (p < 0.001), peaking at 4 h with a period of 28 h. Similar analyses of *BMAL1* (Figure 4F) and *PER2* (Figure 4G) transcripts over 36 h in the hNCI-H716 cells revealed previously reported patterns of expression, similarly validating the synchronization of the cells in this model [17]. *SCGN* also demonstrated strong circadian expression in these cells, peaking at 22 h with a period of 24 h (p < 0.001, Figure 4H).

### 3.4 Temporal interactions of BMAL1 with the *Scgn* promoter

To test whether *Scgn* expression may be driven by BMAL1 binding to the *Scgn* promoter, ChIP analysis was conducted on synchronized mGLUTag cells at time points both before and during the peak and trough of BMAL1 expression and GLP-1 secretion (at 4 and 8 h vs 16 and 20 h). Noncanonical E-boxes were identified in the *Scgn* promoter at 672, 1176, and 1252 bp upstream of the transcription start site (Figure 5A). ChIP analysis (Figure 5B) revealed increased binding at two sites (−672 and −1252 bp) during the peak (4–8 h) compared to the trough (16–20 h) time points (p < 0.05), consistent with a role for BMAL1 in the circadian pattern of *Scgn* expression.

### 3.5 Secretagogin is recruited to the plasma membrane and binds β-actin but not SNAP25 in a temporal manner

To determine whether secretagogin is recruited to the membrane following the stimulation of GLP-1 secretion, the synchronized mGLUTag cells were analyzed by immunocytochemistry to examine the SGCN localization. At the 8-hour time point, increased localization of SCGN at the plasma membrane was observed 60 min after stimulation with the known GLP-1 secretagogue, GIP [16,17] (Figure 6A–B). In contrast, at the 20-hour time point, decreased recruitment was observed 60 min after stimulation (Figure 6C–D).

To establish whether SCGN interacts with the SNARE machinery and/or β-actin in L-cells as previously reported in β-cells [30,32,48], the synchronized mGLUTag cells were treated for 2 h with GIP at both the 8- and 20-hour time points. SNAP25 was then immunoprecipitated (IP) and the blots were probed for co-IP of SCGN (Figure 7A); alternatively, SCGN was pulled-down and the blots were probed for β-actin (Figure 7B). Although SCGN was found to co-IP with SNAP25, no differences in interaction were demonstrated based on the clock time or with GIP stimulation. However, not only was SCGN found to also interact with β-actin, but the amount of β-actin bound to SCGN increased with stimulation at the 8-hour time point only (p < 0.05). Together, these findings are consistent with β-actin's role in SCGN translocation to the plasma membrane during GLP-1 secretion.

### 3.6 Scgn is essential for peak GLP-1 secretion

To investigate the functional importance of secretagogin in GLP-1 secretion, a GLP-1 secretion experiment was conducted in the synchronized mGLUTag cells following knockdown of *Scgn*. Sc- and siRNA treatments had no effect on synchronization, as shown by the expected anti-phasic expression of *Bmal1* and *Per2* at the peak and trough time points (Figure 8A–B). However, secretagogin knockdown was significant at both the mRNA and protein levels at 8 and 20 h (p < 0.05, Figure 8C–D), while the expression of transcripts for other key SNARE proteins was unaffected (Figure S4). A GLP-1 secretion assay was then conducted at the peak (8 h) and trough (20 h) time points under vehicle and GIP-stimulated conditions. Synchronization of the cells was further confirmed by the demonstration of higher GLP-1 secretion in response to GIP at the 8-hour time point compared to the 20-hour time point as previously reported [16,21] in the scRNA-treated cells (p < 0.05, Figure 8E). *Scgn* knockdown had no effect on the basal GLP-1 secretion at either time point. However, Scgn knockdown decreased GLP-1 secretion in response to GIP at the 8-hour time point (p < 0.01) but had no effect at 20 h, demonstrating secretagogin's role in the circadian secretion of GLP-1. This loss of response to GIP was observed although *Gipr* mRNA expression was actually elevated at 20 h compared to 8 h after cell synchronization (Figure S5).

## 4 Discussion

While circadian rhythms are primarily caused by light, peripheral metabolic tissues can be entrained by food intake. As shift workers have a higher incidence of obesity and type 2 diabetes [[49], [50], [51]], these epidemiological data implicate diurnal insulin patterns in disease. The incretin hormones account for approximately 50% of insulin secretion after a meal [52,53], and secretion by the β-cells is coordinated, at least in part, by circadian rhythms in GLP-1 release [16]. However, although GLP-1 secretion by mGLUTag L-cells has been shown to parallel *Bmal1* expression [16], and primary intestinal cultures from Bmal1 KO mice show decreased GLP-1 secretion ex vivo [21], the effect of Bmal1 KO on diurnal GLP-1 secretion in vivo remained unknown. Furthermore, the exact mechanism by which Bmal1 regulates circadian GLP-1 release is also unclear, although our previous research demonstrated roles of thyrotrophic embryonic factor and protein tyrosine phosphatase 4a1 in regulating the peak of GLP-1 release [16]. We have also shown the importance of the Bmal1-nicotinamide phosphoribosyltransferase (NAMPT) pathway, identifying mitochondrial activity and ATP-dependent cellular metabolism as essential for peak GLP-1 secretion [21]. The results of the present study demonstrate that the accessory SNARE protein, secretagogin, is not only a target of Bmal1, but also plays an essential role in regulating the peak, but not the trough, of circadian GLP-1 secretion.

Previous studies of rats identified a circadian rhythm in the GLP-1 secretory response to an oral glucose load that peaked at ZT10, just prior to the onset of their dark or active/feeding period [16]. The mouse model also exhibited circadian GLP-1 secretion in response to the same stimulus; however, the peak of secretion was slightly shifted, occurring at ZT14 as the mice entered their feeding period. These findings in rodents are consistent in that peak GLP-1 secretion in both species occurs as an anticipatory response to increased food intake occurring throughout the dark period. Interestingly, although obesogenic feeding in rats disrupts the rhythm in GLP-1 release such that the normal trough of secretion at the onset of the light period is lost [18], KO of Bmal1 in mice not only disrupted rhythmic GLP-1 release, but also impaired GLP-1 secretion at the peak time point only. Whether these specific differences in timing are consequent to the nature of the circadian disruptors utilized and/or represent species-dependent differences remain unknown. However, similar differences have been noted in humans, wherein a phase delay of 3 × 27 h has been reported to impair GLP-1 release, whereas a 9-hour phase advance has been reported to have no effect [54,55].

Previous studies demonstrated key roles for several SNARE proteins in GLP-1 secretion (VAMP2, STX1A, and SYT7) [[24], [25], [26]]. Given the demonstrated rhythmic expression of SNARE proteins in other endocrine cell types, such as islet α- and β-cells [28], they are ideally situated to provide a mechanistic link between circadian Bmal1 expression and GLP-1 secretion. Furthermore, isolated L-cells from mouse models of elevated GLP-1 secretion demonstrate changes in vesicle organization and vesicle localization [56]. Consistent with this evidence, our transcriptomic and proteomic findings show that the pathways related to both vesicle transport and the SNARE proteins are upregulated at the peak GLP-1 secretion time point. SCGN was also found to co-IP with β-actin as well as with the SNARE protein SNAP25, consistent with findings in other cell types, including β-cells [30,48,57].

Actin cytoskeletal pathways were found to be upregulated in the mGLUTag L-cells at the peak secretory time point by both microarray and mass spectrometry analysis. This is consistent with studies showing that the actin cytoskeleton plays an essential role in hormone secretion by the L-cells and β-cells, wherein actin remodeling upon stimulation is necessary to permit the stimulation of granule exocytosis [34,[58], [59], [60], [61]]. It is therefore possible that secretagogin plays a role in this remodeling to regulate GLP-1 secretion in a temporal manner. In line with this evidence, SCGN has been shown to be important in the organization of the actin cytoskeleton, interacting with trafficking proteins and regulating focal adhesion [32,62]. In the β-cells, this interaction of SCGN with actin has been shown to increase with stimulation [62], a finding that was reproduced in our studies in the L-cells, with stimulation causing increased secretagogin binding to β-actin at the 8-hour time point but not at 20 h. Further investigation is required to determine if the temporal β-actin-secretagogin interaction directly affects GLP-1 secretion. However, interestingly, as the interaction with β-actin was found to be time-dependent, whereas that with SNAP25 was not, these findings suggest an active role for SGCN in the translocation of GLP-1-filled secretory granules to the cell membrane and a more permissive role in SNARE-mediated exocytosis. Further evidence for this is provided by the demonstration that only peak GLP-1 secretion by the mGLUTag L-cells was associated with increased translocation of SGCN to the cell membrane. This is also in agreement with the decreased recruitment of Scgn to the cell membrane observed at the 20-hour time point, which potentially explains the trough GLP-1 secretory response observed in this study and in other publications [[16], [17], [18],^21^].

Expressed in a rhythmic manner in both the mGLUTag and hNCI-H716 cells, *Scgn* transcript levels paralleled those of SCGN with a 4-hour translational delay. The Scgn expression patterns were also more consistent with those of Bmal1 in both the murine and human cells, rather than with that of *Per2*, which was anti-phasic to *Bmal1* and *Scgn* in the mGLUTag L-cells, but arrhythmic in the hNCI-H716 L-cells (present study and [16,17]). These findings further confirmed Bmal1 as a prime target regulating circadian GLP-1 secretion, as was also demonstrated by ChIP analysis, demonstrating that the *Scgn* promoter is a direct target of BMAL1, with greater binding of BMAL1 to 2 E-boxes in *Scgn* at the peak compared to the trough time point of GLP-1 secretion. Interestingly, in vivo loss of Bmal1 and in vitro knockdown of Scgn resulted in a similar phenotype of loss in GLP-1 secretory rhythm and impaired secretion only at the peak time point, further suggesting a tight interplay between Bmal1 and Scgn. Furthermore, consistent with an essential role of SGCN in circadian GLP-1 secretion, only peak GLP-1 release was reduced by *Scgn* knockdown. Interestingly, this effect was observed only with respect to the stimulated GLP-1 release at this time point, with no observed change in the basal secretion. These findings are consistent with studies showing that disruption of the actin cytoskeleton and syntaxin-1A knockout cause impairments solely in stimulated compared to basal GLP-1 secretion [25,34]. Similar studies of NIT-1 β-cells also showed that only second-phase insulin secretion was altered following *Scgn* silencing [32]. Indeed, cell stimulation is known to be essential for SGCN's role as a calcium sensor, inducing conformational changes that are required for the facilitation of secretion [[29], [30], [31],^48^,^62^,^63^]. In the β-cells, this appears to be due, at least in part, to inhibition by tomosyn, which dissociates from SCGN when exposed to calcium, liberating SCGN to interact with its secretory partners, including actin and SNAP25 [30]. Consistent with this, the secretagogue used in the present study, GIP, increases the cAMP levels in the L-cells, which is known to enhance intracellular Ca^2+^ concentrations and lead to increased GLP-1 secretion [64]. Finally, other SNARE regulators that have been suggested to be rhythmic [28] and are known to be essential for insulin secretion [65,66] were also identified in the mGLUTag L-cell proteome as being upregulated at the peak secretion time point, including the SNARE-accessory protein STXBP-1 (Munc-18). Further investigation into the role of other potential mediators of circadian GLP-1 secretion is thus warranted.

Importantly, SCGN was identified in all primary murine and human L-cells. Given recent evidence demonstrating a diversity of L-cells along the crypt villus axis [67], L-cell SCGN expression appears to be ubiquitous, suggesting that it plays an essential role in L-cell function. Secretagogin may also influence the development of L-cells, as evidence shows that it plays an essential role in β-cell development and causes α-cell hyperplasia when knocked out [33]. Recent reports also implicate reduced secretagogin expression in type 2 diabetes [[68], [69], [70]], consistent with a report that whole body SCGN KO mice are glucose intolerant [69]. Given the importance of GLP-1 and, subsequently, insulin secretion for the maintenance of glucose homeostasis, it is possible that some of the effects of altered secretagogin expression may be L-cell mediated.

It is acknowledged that the Scgn knockdown studies presented herein were only conducted in vitro. Although the cellular models utilized are known to be representative of primary L-cells in terms of their GLP-1 secretion and response to secretagogues [16,21,35,71], one drawback to in vitro circadian experiments is that in vivo rhythms are orchestrated by a range of cues, with L-cells synchronized by nutrient intake and a variety of hormones, neural inputs, cytokines, and the microbiome. Thus, in vitro experiments are subject to a slow loss of synchronicity given the absence of such cues, limiting experiments to 48-hour time intervals. Nonetheless, while our cells may lack these cues, synchronization protocols have been established to mimic these environments, driving these rhythms as seen in other tissues and cell types [[16], [17], [18],^21^,^27^,^28^,^72^,^73^]. Additionally, our studies used only single synchronization protocol (i.e., with forskolin), although studies of β-cells have shown that multiple different synchronizing agents that also act through the cAMP-PKA pathway generate similar effects on the circadian expression of a *Per2-luciferase* construct [36]. Finally, GIP was used as the sole secretagogue in the present study, suggesting that the results may apply solely to GIP-stimulated GLP-1 secretion; however, previous studies directly compared the temporal responses to GIP with those of several other L-cell secretagogues (insulin and bethanechol) in the mGLUTag L-cells, finding no differences between the 3 agents with respect to the peak and trough GLP-1 secretory responses [[16], [17], [18],^21^].

In conclusion, we have established the circadian pattern of GLP-1 secretion in mice and shown that it is dependent on the core clock gene Bmal1. We have also characterized secretagogin expression in murine and human L-cells for the first time, identifying secretagogin as a novel regulator of circadian GLP-1 secretion: under the control of rhythmic BMAL1 expression, SCGN binds to β-actin, is recruited to the cell membrane in a time-dependent manner that is parallel to its circadian expression and is required for stimulated-GLP-1 secretion. Due to the insulinotropic effects of GLP-1, both long-acting GLP-1 receptor agonists and GLP-1 degradation inhibitors are used as a therapy for type 2 diabetes [74]. SNARE regulators may provide a novel target for type 2 diabetes therapies given their regulation of the secretion of key metabolic hormones, including not only GLP-1, but also insulin. Further investigation of the developing link between circadian rhythms, diabetes, and obesity [[49], [50], [51]] and improved understanding of the molecular drivers behind the circadian secretion of GLP-1 may therefore have therapeutic implications, including time-sensitive therapies.

## Contributions

ADB and AM designed and conducted the studies, performed the analyses, and wrote the paper. EM, PG, EM, JC, AD, JCM, AEA, FR, FMG, and MG-L conducted the studies and performed the analyses. BJC conducted the analyses. PLB designed the studies, conducted the analyses, and wrote the paper. All authors approved the final manuscript.

## Disclosures

ADB, AM, EM, PG, EM, JC, AD, JCM, AEA, FR, FMG, MG-L, and PLB have nothing to disclose.
