# Supplementary material for: The core clock gene, Bmal1, and its downstream target, the SNARE regulatory protein secretagogin, are necessary for circadian secretion of glucagon-like peptide-1
Source: Mol Metab. 2019 Nov 21;31:124–37. doi: 10.1016/j.molmet.2019.11.004 (PMC6920326; doi:10.1016/j.molmet.2019.11.004)
Supplement: Multimedia component 1 [file mmc1.docx]

# Supplemental Tables and Figures

**Supplemental Table 1 – TaqMan Primers**

| **Primer** | **Product Code** | **Species** |
| --- | --- | --- |
| *Scgn* | Mm00520475_m1 | Mouse |
| *Arntl* | Mm00500226_m1 | Mouse |
| *Per2* | Mm00478113_m1 | Mouse |
| *H3f3a* | Mm01612808_g1 | Mouse |
| *Actb* | Mm02619580_g1 | Mouse |
| *Snap25* | Mm01276449_m1 | Mouse |
| *Vamp2* | Mm01325243_m1 | Mouse |
| *Stx1a* | Mm00444008_m1 | Mouse |
| *Stxbp1* | Mm00436837_m1 | Mouse |
| *Gipr* | Mm01316344_m1 | Mouse |
| *Scgn* | Hs00199630_m1 | Human |
| *Arntl* | Hs00154147_m1 | Human |
| *Per2* | Hs00256143_m1 | Human |
| *Hist1h3a* | Hs00543854_s1 | Human |

**Supplemental Table 2 – PCR Primers for ChIP**

| **Primer Site** | **Direction (5’ to 3’)** | **Sequence** |
| --- | --- | --- |
| 672 | AAATGGTGGCTGCCAATTTG | Forward |
| 672 | AAACCTAGCCACACTGTATC | Reverse |
| 1176 | AGAGACCTAGAATAGTGACC | Forward |
| 1176 | ATTCGTAAGGAGCAACAGAG | Reverse |
| 1252 | AGAACACAGTCATTCCGTCA | Forward |
| 1252 | TAACGAAGAGTACAATGTCT | Reverse |

**Supplemental Table 3 – Antibodies**

| **Analysis** | **Primary Antibody** | **Dilution** | **RRID** | **Secondary Antibody** | **Dilution** | **RRID** |
| --- | --- | --- | --- | --- | --- | --- |
| **Tissue Immunofluorescence** | Rabbit anti-SCGN | 1:250 | AB_2798371 | Anti-rabbit IgG HRP-linked | 1:150 | AB_2099233 |
|  | Mouse anti-GLP-1 | 1:400 | AB_447455 | Anti-mouse IgG HRP-linked | 1:150 | AB_330924 |
| **Cellular Immunofluorescence** | Rabbit anti-SCGN | 1:250 | AB_2798371 | Anti-rabbit IgG HRP-linked | 1:150 | AB_2099233 |
| **Immunoblot** | Rabbit anti-SCGN | 1:5000 | AB_2798371 | Anti-rabbit IgG HRP-linked | 1:7500 | AB_2099233 |
|  | Rabbit anti-BMAL1 | 1:1000 | AB_2728705 | Anti-rabbit IgG HRP-linked | 1:7500 | AB_2099233 |
|  | Rabbit anti-H3A | 1:1000 | AB_2118461 | Anti-rabbit IgG HRP-linked | 1:7500 | AB_2099233 |
|  | Rabbit anti- β-actin | 1:5000 | AB_476693 | Anti-rabbit IgG HRP-linked | 1:7500 | AB_2099233 |
| **Chromatin-Immunoprecipitation** | Rabbit anti-BMAL1 | 1:50 | AB_2728705 |  | | |
| **Immunoprecipitation** | Rabbit anti-SCGN | 1:50 | AB_2798371 |  |  |  |
|  | Anti-rabbit SNAP25 | 1:50 | AB_887790 |  |  |  |

**Supplemental Table 4 –Microarray: significantly changed genes**

| **Symbol** | **logFC** | **Adjusted**  **p-value** |
| --- | --- | --- |
| *ARHGAP8* | 1.4275051 | 0.02681398 |
| *NKX6-2* | 1.3479209 | 0.02727856 |
| *FIBCD1* | 1.2822624 | 0.02727856 |
| *INSL5* | 1.3447974 | 0.02824844 |
| *ID1* | 1.2381321 | 0.02824844 |
| *NACC2* | 1.2945363 | 0.02824844 |
| *NXF7* | 1.1567265 | 0.03540702 |
| *PKIB* | 1.3157166 | 0.04397717 |
| *TMEM65* | 1.1278556 | 0.04397717 |
| *SSTR2* | 1.1040827 | 0.04549345 |
| *GPX2* | 1.0575818 | 0.04549345 |
| *ANKRD6* | 1.2002185 | 0.04557098 |
| *ARNTL* | 1.1032882 | 0.04557098 |

**Supplemental Table 5 – Microarray: Gene Ontology enriched pathways relating to secretion**

| **GO Pathway** | **# of genes** | **Direction** | **p- value** |
| --- | --- | --- | --- |
| GO_RETROGRADE_VESICLE_MEDIATED_TRANSPORT_GOLGI_TO_ER | 155 | Up | 5.41E-09 |
| GO_INTRA_GOLGI_VESICLE_MEDIATED_TRANSPORT | 73 | Up | 3.65E-07 |
| GO_POST_GOLGI_VESICLE_MEDIATED_TRANSPORT | 206 | Up | 4.78E-07 |
| GO_SNARE_COMPLEX (see Fig. S1) | 90 | Up | 1.14E-06 |
| GO_GOLGI_VESICLE_TRANSPORT | 704 | Up | 1.66E-06 |
| GO_COPI_COATED_VESICLE | 49 | Up | 4.74E-05 |
| GO_ER_TO_GOLGI_VESICLE_MEDIATED_TRANSPORT | 383 | Up | 8.58E-05 |
| GO_GOLGI_ASSOCIATED_VESICLE | 188 | Up | 1.66E-04 |
| GO_VESICLE_CYTOSKELETAL_TRAFFICKING | 93 | Up | 2.76E-04 |
| GO_VESICLE_DOCKING | 86 | Up | 2.99E-04 |
| GO_GOLGI_ASSOCIATED_VESICLE_MEMBRANE | 103 | Up | 8.57E-04 |
| GO_RETROGRADE_TRANSPORT_VESICLE_RECYCLING_WITHIN_GOLGI | 31 | Up | 1.06E-03 |
| GO_SYNAPTIC_VESICLE_CYTOSKELETAL_TRANSPORT | 17 | Up | 1.15E-03 |
| GO_ANTEROGRADE_SYNAPTIC_VESICLE_TRANSPORT | 17 | Up | 1.15E-03 |
| GO_SYNAPTIC_VESICLE_TRANSPORT_ALONG_MICROTUBULE | 17 | Up | 1.15E-03 |
| GO_COPI_COATED_VESICLE_MEMBRANE | 32 | Up | 1.26E-03 |
| GO_COPI_VESICLE_COAT | 25 | Up | 1.28E-03 |
| GO_VESICLE_COAT | 97 | Up | 1.32E-03 |
| GO_VESICLE_DOCKING_INVOLVED_IN_EXOCYTOSIS | 62 | Up | 2.14E-03 |
| GO_VESICLE_ORGANIZATION | 585 | Up | 3.38E-03 |
| GO_VESICLE_COATING | 184 | Up | 4.08E-03 |
| GO_TRANS_GOLGI_NETWORK_TRANSPORT_VESICLE | 76 | Up | 5.05E-03 |
| GO_METANEPHRIC_RENAL_VESICLE_MORPHOGENESIS | 19 | Down | 7.08E-03 |
| GO_VESICLE_TARGETING | 209 | Up | 7.48E-03 |
| GO_VESICLE_LOCALIZATION | 550 | Up | 9.04E-03 |
| GO_CLATHRIN_SCULPTED_VESICLE | 28 | Up | 9.63E-03 |
| GO_COATED_VESICLE_MEMBRANE | 308 | Up | 1.33E-02 |
| GO_COATED_VESICLE | 539 | Up | 1.37E-02 |
| GO_VESICLE_MEDIATED_TRANSPORT_BETWEEN_ENDOSOMAL_COMPARTMENTS | 59 | Up | 1.37E-02 |
| GO_CLATHRIN_VESICLE_COAT | 63 | Up | 1.48E-02 |
| GO_CLATHRIN_COATED_VESICLE | 332 | Up | 1.49E-02 |
| GO_ACTIN_CYTOSKELETON (see Fig. S2) | 908 | Up | 1.79E-02 |
| GO_VESICLE_MEDIATED_TRANSPORT | 2522 | Up | 1.84E-02 |
| GO_EXOCYTIC_VESICLE | 352 | Up | 2.05E-02 |
| GO_TRANSPORT_VESICLE (see Fig. 1A) | 811 | Up | 4.12E-02 |
| GO_CLATHRIN_COAT_OF_TRANS_GOLGI_NETWORK_VESICLE | 28 | Up | 4.63E-02 |

**Supplemental Figure 1**

**Supplemental Figure 1**: **Circadian patterns of insulin and blood glucose in C57Bl/6J and Bmal1 WT and KO mice.** (**A**) Plasma levels of insulin and blood glucose were assessed by OGTT in 4-hour fasted C57Bl/6J mice throughout the 24 hour light (open bars)-dark (closed bars) cycle (ZT0 = 06:00). (**B-C**) Plasma levels of insulin and blood glucose as well as the accompanying ΔAUC for 4-hour fasted Bmal1 WT and KO mice after an OGTT at ZT2 and ZT14 are represented in (**B**) for insulin and (**C**) for blood glucose **(**n = 4-8 mice for all time points in all experiments). t_0_ indicates the absolute fasting values for insulin or glycemia, as appropriate, in pg/ml and mM, respectively.* p < 0.05

**Supplemental Figure 2**

**Supplemental Figure 2: Microarray GO SNARE complex.** Heat map of GO SNARE Complex pathway analyzed by microarray in synchronized mGLUTag L-cells, showing log_2_fold-change between the two time points, 4 and 16 hours (n = 3 for each time point). p < 0.05

**Supplemental Figure 3**


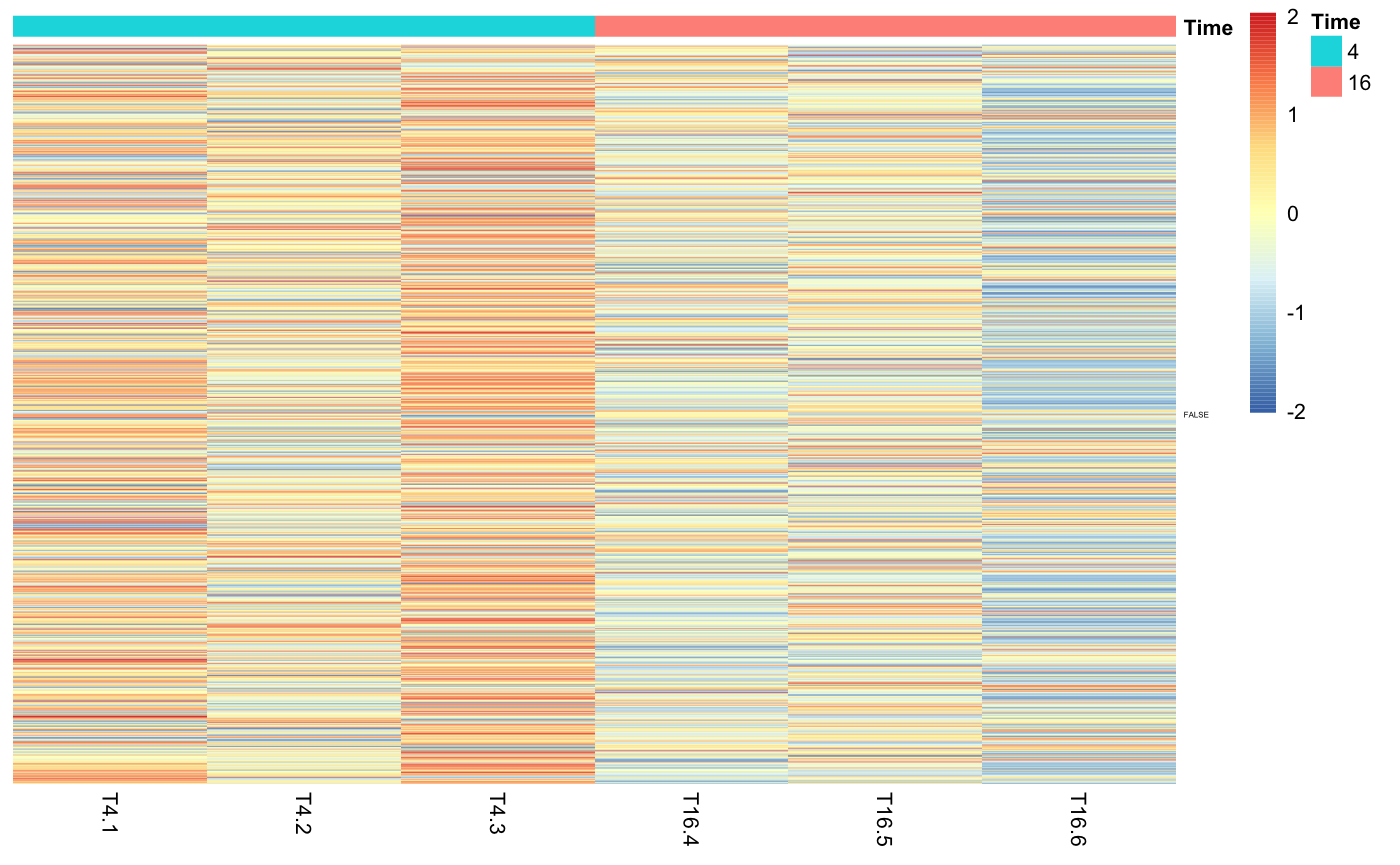


**Supplemental Figure 3: Microarray: GO actin cytoskeleton.** Heat map of the GO Actin Cytoskeleton pathway analyzed by microarray in synchronized mGLUTag L-cells, showing log_2_fold-change between the two time points, 4 and 16 hr (n=3 for each time point). p < 0.05

**Supplemental Figure 4**

**Supplemental Figure 4: SNARE protein mRNA expression in Scgn knockdown cells.** qRT-PCR analysis of SNARE protein mRNA expression in untreated, sc-RNA and Scgn si-RNA mGLUTag cells (n=4 for each treatment; conducted as 2 replicates from each of 2 independent splits).

**Supplemental Figure 5**

**Supplemental Figure 5: GIPR mRNA expression in Scgn knockdown cells.** qRT-PCR analysis of GIPR mRNA expression in untreated, sc-RNA and Scgn si-RNA mGLUTag cells (n=4 for each treatment; conducted as 2 replicates from each of 2 independent splits). *p < 0.05, ***p < 0.001
